# The case for not restricting saturated fat on a low carbohydrate diet

**DOI:** 10.1186/1743-7075-2-21

**Published:** 2005-08-31

**Authors:** Jeff S Volek, Cassandra E Forsythe

**Affiliations:** 1 Human Performance Laboratory, Department of Kinesiology, University of Connecticut, Storrs, CT, USA

We would like to compliment Drs. Arora and McFarlane on their timely review of low carbohydrate diets in diabetes management [1]. Undeniably, the prescription of low-fat, high-carbohydrate diets to treat diabetes has to be questioned and the power of carbohydrate restriction seriously considered. The article dispels common myths and provides a convincing argument for successful use of carbohydrate restriction in treating diabetes. One point stressed by Arora and McFarlane was that mono and polyunsaturated fat should be emphasized over saturated fat as a way to achieve caloric balance on a carbohydrate-restricted diet. We contend that the recommendation to intentionally restrict saturated fat is unwarranted and only serves to contribute to the misleading rhetoric surrounding the health effects of saturated fat.

We believe restriction of saturated fat is not warranted on a low-carbohydrate diet because of our work showing favorable responses in clinical risk factors for diabetes and cardiovascular disease in low-carbohydrate diets that were rich in saturated fat [2]. In addition, German & Dillard [3] have reviewed several experimental studies of the effects of saturated fats and the results are found to be variable and there is a general failure to meet the kind of unambiguous predictions that would justify the recommendation to reduce saturated fat in the population [3]. Other critical reviews of the evidence [4] have questioned whether public health recommendations for reducing saturated fat intake [5] are appropriate.

The critical issues are:

1. The atherogenic potential of saturated fats varies greatly depending on chain length and whether it is present alone or added in foods. Stearic acid (C18) is a major saturated fat found in beef, chicken, and pork and has repeatedly been shown not to raise LDL cholesterol levels [6]. Even palmitic acid (C16), the most abundant saturated fatty acid in the diet, does not raise LDL cholesterol in the presence of adequate linoleic acid [7].

2. The effect of saturated fat cannot be assumed to be independent of specific dietary conditions. In particular, hypocaloric or low total fat diets may show different results than deduced from epidemiology. A recent report [8] showed that for a woman on a relatively low fat diet, a greater saturated fat intake was associated with a reduced progression of coronary atherosclerosis. An editorial described this as "an American paradox [9].

3. Evaluation of the overall health effects of saturated fat requires consideration of markers in addition to LDL-cholesterol. Isocaloric replacement of carbohydrate with any type of fat results in decreased triglycerides and increased HDL-cholesterol, the effect on HDL-cholesterol being greater for saturated fat compared to unsaturated fat [10]. Reductions in saturated fat also adversely affect HDL subpopulations by decreasing larger HDL_2_-cholesterol concentrations [11], whereas increases in saturated fat increase this antiatherogenic fraction [12,13]. Furthermore, very low-carbohydrate diets rich in saturated fat increase LDL size and conversion from a high-risk pattern B to a lower risk pattern A phenotype [2].

4. Finally, there is the concern that recommendations to limit saturated fat would lead to their replacement with carbohydrate, which can have undesirable effects (increased triglycerides with decreased HDL cholesterol) [10].

For these reasons, we believe that the recommendation to restrict saturated fat in favor of unsaturated fat on a low-carbohydrate diet is unnecessary and may even diminish some of the beneficial physiological effects associated with carbohydrate restriction. At the very least, the food restriction required to reduce saturated fat will compromise the palatability of the diet and ultimately the acceptance of the approach to diabetes management recommended by Arora and McFarlane [1].

## References

[B1] Arora SK, McFarlane SI (2005). The case for low carbohydrate diets in diabetes management. Nutr Metab (Lond).

[B2] Volek JS, Sharman MJ, Forsythe CE (2005). Modification of lipoproteins by very low-carbohydrate diets. J Nutr.

[B3] German JB, Dillard CJ (2004). Saturated fats: what dietary intake?. Am J Clin Nutr.

[B4] Ravnskov U (1998). The questionable role of saturated and polyunsaturated fatty acids in cardiovascular disease. J Clin Epidemiol.

[B5] Trumbo P, Schlicker S, Yates AA, Poos M (2002). Dietary reference intakes for energy, carbohydrate, fiber, fat, fatty acids, cholesterol, protein and amino acids. J Am Diet Assoc.

[B6] Grundy SM (1994). Influence of stearic acid on cholesterol metabolism relative to other long-chain fatty acids. Am J Clin Nutr.

[B7] French MA, Sundram K, Clandinin MT (2002). Cholesterolaemic effect of palmitic acid in relation to other dietary fatty acids. Asia Pac J Clin Nutr.

[B8] Mozaffarian D, Rimm EB, Herrington DM (2004). Dietary fats, carbohydrate, and progression of coronary atherosclerosis in postmenopausal women. Am J Clin Nutr.

[B9] Knopp RH, Retzlaff BM (2004). Saturated fat prevents coronary artery disease? An American paradox. Am J Clin Nutr.

[B10] Katan MB, Zock PL, Mensink RP (1995). Dietary oils, serum lipoproteins, and coronary heart disease. Am J Clin Nutr.

[B11] Berglund L, Oliver EH, Fontanez N, Holleran S, Matthews K, Roheim PS, Ginsberg HN, Ramakrishnan R, Lefevre M (1999). HDL-subpopulation patterns in response to reductions in dietary total and saturated fat intakes in healthy subjects. Am J Clin Nutr.

[B12] Hays JH, DiSabatino A, Gorman RT, Vincent S, Stillabower ME (2003). Effect of a high saturated fat and no-starch diet on serum lipid subfractions in patients with documented atherosclerotic cardiovascular disease. Mayo Clin Proc.

[B13] Seshadri P, Iqbal N, Stern L, Williams M, Chicano KL, Daily DA, McGrory J, Gracely EJ, Rader DJ, Samaha FF (2004). A randomized study comparing the effects of a low-carbohydrate diet and a conventional diet on lipoprotein subfractions and C-reactive protein levels in patients with severe obesity. Am J Med.

